# Cell-free protein synthesis from non-growing, stressed *Escherichia coli*

**DOI:** 10.1038/s41598-017-16767-7

**Published:** 2017-11-28

**Authors:** Jurek Failmezger, Michael Rauter, Robert Nitschel, Michael Kraml, Martin Siemann-Herzberg

**Affiliations:** Institute of Biochemical Engineering, University of Stuttgart, Stuttgart, Germany

## Abstract

Cell-free protein synthesis is a versatile protein production system. Performance of the protein synthesis depends on highly active cytoplasmic extracts. Extracts from *E. coli* are believed to work best; they are routinely obtained from exponential growing cells, aiming to capture the most active translation system. Here, we report an active cell-free protein synthesis system derived from cells harvested at non-growth, stressed conditions. We found a downshift of ribosomes and proteins. However, a characterization revealed that the stoichiometry of ribosomes and key translation factors was conserved, pointing to a fully intact translation system. This was emphasized by synthesis rates, which were comparable to those of systems obtained from fast-growing cells. Our approach is less laborious than traditional extract preparation methods and multiplies the yield of extract per cultivation. This simplified growth protocol has the potential to attract new entrants to cell-free protein synthesis and to broaden the pool of applications. In this respect, a translation system originating from heat stressed, non-growing *E. coli* enabled an extension of endogenous transcription units. This was demonstrated by the sigma factor depending activation of parallel transcription. Our cell-free expression platform adds to the existing versatility of cell-free translation systems and presents a tool for cell-free biology.

## Introduction

Cell-free transcription and translation systems have emerged as powerful toolboxes for systems and synthetic biology approaches^1–3^. What began decades ago as a tool for understanding polypeptide synthesis^4^ is now made up of up-to-date *in vitro* translation systems, a versatile technique to express proteins and to understand and create biological networks^5–8^.

Cell-free protein synthesis (CFPS) systems comprise a large repertoire of biochemical pathways that can easily be controlled and manipulated^9^. Recent examples are (i) the directed incorporation of non-canonical amino acids into proteins at multiple sites^6^, (ii) the construction and characterization of multiple genetic circuits^2^, and (iii) the engineering of artificial minimal cell systems^10–12^ such as phospholipid vesicles containing the entire translation machinery. These artificial environments are designed to potentially perform multifaceted biological tasks such as controlled exchange of nutrients^3^.

Among many available crude extract cell-free expression systems derived from either eukaryotic or prokaryotic cells, the *Escherichia coli* system is still the most popular^13^. Designed as a coupled transcription and translation system, transcription is usually performed by supplementing the reaction with the highly specific and efficient bacteriophage T7 RNA polymerase^14^. More-recent approaches demonstrate the use of endogenous *E. coli* RNA polymerase and “housekeeping” σ^70^ as a strong transcription unit to produce proteins *in vitro* ^15^. This setup allows for an expansion of transcription regulatory parts and has proven to be extremely suitable, with its application ranging from highly efficient protein production^16^ to prototyping of gene circuits^17^.

A prerequisite for efficient protein translation is a steady supply of energy. Therefore, cell-free translation systems are engineered to regenerate ATP to fuel translation^18^. Whereas using phosphorylated energy donors results in inorganic phosphate accumulation and sequestration of magnesium ions, a serious limitation that triggers rRNA cleavage and ribosome breakdown as we have previously shown^19^, other energy sources such as pyruvate allow extended reaction homeostasis. Therefore, recruiting the complete metabolic scheme of the glycolytic pathway lead to advanced long lasting reaction systems and high final product titers^20–22^.

Despite recent developments in *in vitro* reaction design, cell-free translation systems heavily rely on the active translation machinery usually derived from cytoplasmic extracts (S30 extract). The well-accepted standard procedure for extract preparation, consisting of cell cultivation, cell lysis, and run off ^23^, has remained largely unchanged^24,25^. Current procedures suggest a cell harvest during the early logarithmic growth phase^26–28^, given that fast-growing cells contain high intracellular concentrations of ribosomes and other components necessary for efficient translation^29^. The major drawback, however, is the low yield of cell-free extract per initial culture volume and the inefficient use of culture broth. Furthermore, cultivation of cells is time consuming and monitoring of exponential growth is laborious. Moreover, high versatility of genetic endogenous regulatory mechanisms is required when using cell-free expression systems^3^. The currently available regulatory mechanisms are constrained by the physiological background of the biomass at the time of cell harvest (fast growth). For example, with only one sigma factor present in the cell-free extract, transcription modularity is still poor^2^. Therefore, expanding the range of potential regulatory networks and transcription modules in cell-free translation systems is required.

In the present study, we demonstrate that cell-free extracts derived from non-growing and stressed *E. coli* cells cultivated over night are active, which was previously considered impossible. We also systematically characterize the translation machinery of cell-free extracts obtained from stressed and non-stressed conditions. We hope that our study highlights the versatility and suitability of an expression system derived from non-growing, stressed cells as a potential tool for cell-free protein synthesis.

## Results and Discussion

### Assessment of cell-free extracts from growing and non-growing, stressed cells

**Figure 1:**
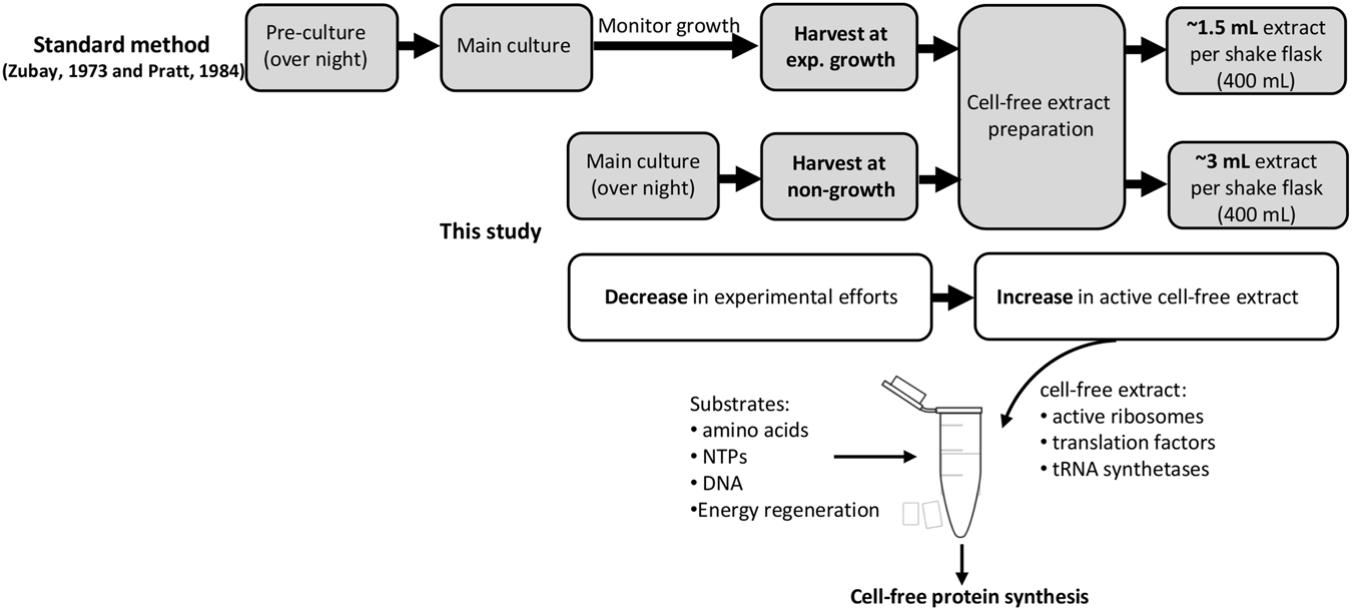
Schematic comparison of the standard cell-free extract preparation method and the simplified approach presented in the current study.

**Figure 2:**
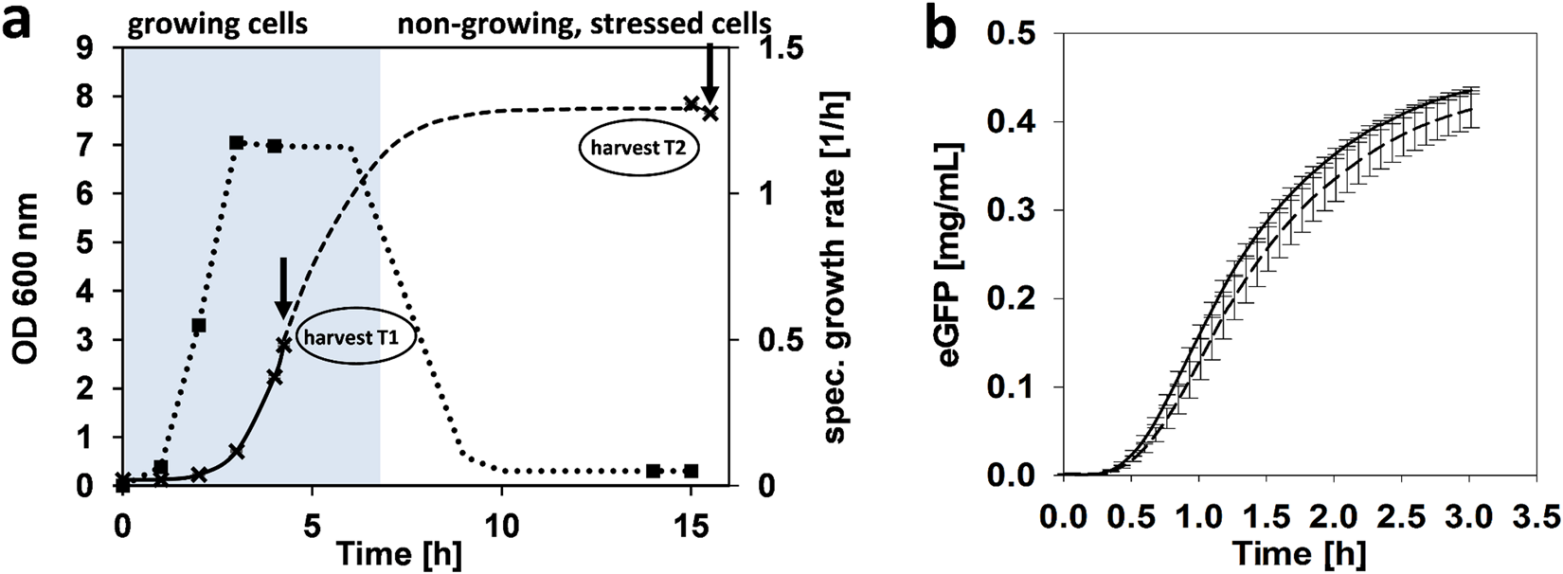
Active cell-free expression derived from stressed *Escherichia coli* cells. (**a**) Scheme of cultivation. Time course of cell growth (dashed line) and specific growth rate (dotted line). T1 denotes the time of harvest during fast growth and T2 denotes the time of harvest at non-growth, stressed conditions. (**b**) Time course of cell-free protein expression of eGFP. Expression based on cell-free extract of resting cells (solid line) and fast-growing cells (dotted line). Expression was assessed at 250 µL scale at 37 °C (n = 3).

In contradiction to current protocols that suggest a rather narrow window for cell-harvest at exponential and fast growth, the goal of this study was to test whether cells at stationary phase conditions allow producing active cell-free extract (Fig. 1). This would enhance the diversity of possible applications of CFPS systems. First, *E. coli* A19 was cultivated in a shaking flask at 37 °C in 2 × YTPG medium and cells were harvested during the mid-logarithmic growth phase (OD_600_ ≈ 3), which is the recommended point of harvest in current cell-free extract preparation protocols (Fig. 2a). High specific growth rates (1–1.2 h^−1^) are linked to highly active molecular machineries such as ribosomes and translation factors^29,30^. Second, cells were harvested after 15 h of cultivation (over night). No growth was observed at this point, indicating that the cells had entered the stationary phase (Fig. 2a). The biomass from both points of harvest were subjected to cell-free extract preparation according to the standard protocol^23^ with some modifications as previously described by Liu *et al*.^24^.

For comparison, the productivity of extracts prepared from cells under growth and non-growth conditions was assayed in batch reactions. We used eGFP as a model protein under the control of a T7 promoter. Accordingly, cell-free translation reactions were supplemented with T7 RNA polymerase. This transcription unit is independent of any endogenous RNA polymerase activity present in the crude extracts and, therefore, transcription is hardly influenced by a possible divergence of the metabolic characteristics of cell-free extracts. Besides certain drawbacks of eGFP, for example it was reported that only a minor fraction of the *in vitro* synthesized reporter protein is active^31^, eGFP offers the advantage of measuring cell-free protein synthesis online. Hence, conclusions about the system performance can be drawn immediately. To allow for sampling, reactions were performed in a rather large scale of 250 µL, although it is evident that the scale up of the reaction volume impacts performance levels (Supplemental Fig. S1). Thus, for the sake of analysis and system characterization, lower performance levels were accepted. A key to efficient translation is the regeneration of ATP and GTP, both of which are consumed during the translation and transcription reaction. Although cytoplasmic enzymes present in the cell-free extract can be recruited to regenerate ATP^32^, we decided to use an exogenous energy regeneration system consisting of 60 mM creatine phosphate in combination with creatine kinase^33^. This allows the regeneration of ATP to be unaffected by the nature of the tested extracts, a prerequisite for a comparison of the translation performance. Strikingly, extracts from growing and non-growing cells performed equally well in cell-free translation reactions of eGFP (Fig. 2b).

### Characterization of the translation machinery

Given that bacteria dramatically modify their cellular metabolism when entering the stationary phase (for reviews see^34^ and^35^), our observation that high-active cell-free extract can be obtained from stationary, stressed cells is astonishing. Among the various changes to metabolism, such as alterations in DNA structure and shut down of several metabolic pathways, the translation machinery is highly affected under non-growth conditions. For example, in a previous work, we showed that ribosomes are quickly degraded when cells enter the stationary phase^36^. Other reports have shown that ribosomes are switched off by the cell by initiating a process called ribosome hibernation^37,38^, where inactive ribosome dimers (100 S) are formed from 70 S monomers; these dimers cannot participate in translation^39^.

Here, we obtained a high-active cell-free translation system from cells that, in theory, are prone to stationary phase regulatory mechanisms such as ribosome breakdown. Because this contradicts current opinions, we performed a thorough characterization of the cell-free extract generated from resting cells. Since ribosomes are the main parameter in cell-free translation systems, we assessed the ribosome concentration in extracts from growing and non-growing *E. coli*. We first extracted the total RNA and then determined the concentration of rRNA (sum of 16 S and 23 S rRNA) by capillary gel electrophoresis with laser-induced fluorescence detection (CGE-LIF)^19^. We also measured the total protein concentration. The data in Table 1 imply an approximately twofold reduction of the ribosome (rRNA) content in extracts from non-growing cells. As non-growing cells reduce their rRNA levels^35,36,40^ it appears that this reduced rRNA concentration is a result of ribosome breakdown. Total protein concentrations were also reduced under non-growth conditions, but this reduction was less pronounced. Taken together, we found a substantial alteration in the rRNA/protein ratios of extracts from different *in vivo* backgrounds. Nevertheless, in contrary to other studies that detected a fragmentation of rRNA in stationary *E. coli* ^41^, the CGE-LIF analysis showed only intact 16 S and 23 S rRNA (Supplemental Fig. S2), suggesting that rRNA integrity is conserved.

**Table 1:** rRNA and total protein concentration in cell-free extracts^a^.

Time of harvest	Total protein [mg/mL]	Total rRNA [mg/mL]	Ratio of rRNA/protein
Harvest T1	22.78 ± 3.19	19.62 ± 2.32	0.86 ± 0.15
Harvest T2	16.65 ± 1.23	9.08 ± 1.33	0.55 ± 0.09

^a^rRNA (sum of 16 S and 23 S rRNA) was extracted and quantified by CGE-LIF based on an added internal standard. Total protein concentration was obtained using a colorimetric assay (Pierce BCA protein assay). Shown are means and standard deviations of two independently prepared extracts.

To test whether 100 S ribosomes are present in the cell-free extract from resting cells, we compared the ribosome profiles from both cultivation conditions and those from a positive control.

**Figure 3:**
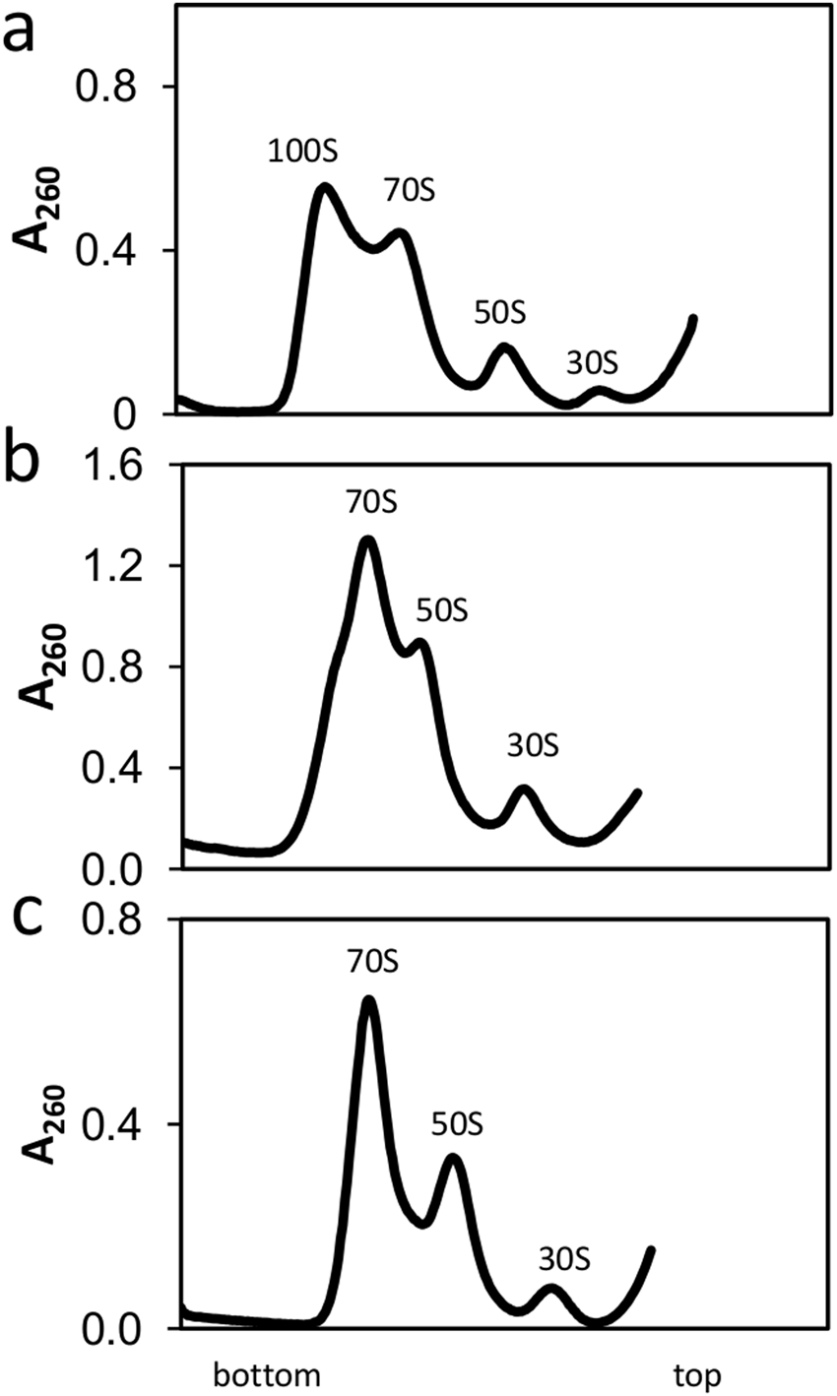
Ribosome profile analysis. (**a**) Positive control of ribosome dimerization and 100 S formation. Here, *E. coli* W3110 was cultivated as described by El-Sharoud and Niven^37^. (**b**) Ribosome profile of cell-free extract from cells harvested during fast growth. (**c**) Ribosome profile of cell-free extract from cells harvested at non-growth.

As can be seen in Fig. 3, no 100 S ribosomes could be detected in the cell-free extract from non-growing cells under the conditions tested. We noticed a drop in the pH at extended cultivations in 2 × YTPG medium, most likely due to acetate formation by *E. coli*. Growth in an acidified medium results in a downshift in the expression of a small protein called ribosome modulation factor (RMF), which is required for 100 S ribosome formation^37^. To elucidate whether the pH drift was responsible for inhibiting 100 S ribosome formation, we omitted glucose during cultivation (2 × YTP medium). Although the pH remained constant during the 15 h of cultivation, no 100 S ribosomes were observed (Supplemental Fig. S3a). Interestingly, cell-free extract from cells cultivated in 2 × YTP medium and harvested at stationary phase was also active (Supplemental Fig. S4). To rule out the possibility that *E. coli* A19 was deficient in 100 S ribosome formation, the bacteria were cultivated in a mineral medium. We observed a slight appearance of 100 S particles (Supplemental Fig. S3b). These results highlight that in our original experiment (2 × YTPG medium), the ribosomes were not modulated to their inactive forms. Nevertheless, it cannot be ruled out that ribosome modulation takes place at a later stage of cultivation.

**Figure 4:**
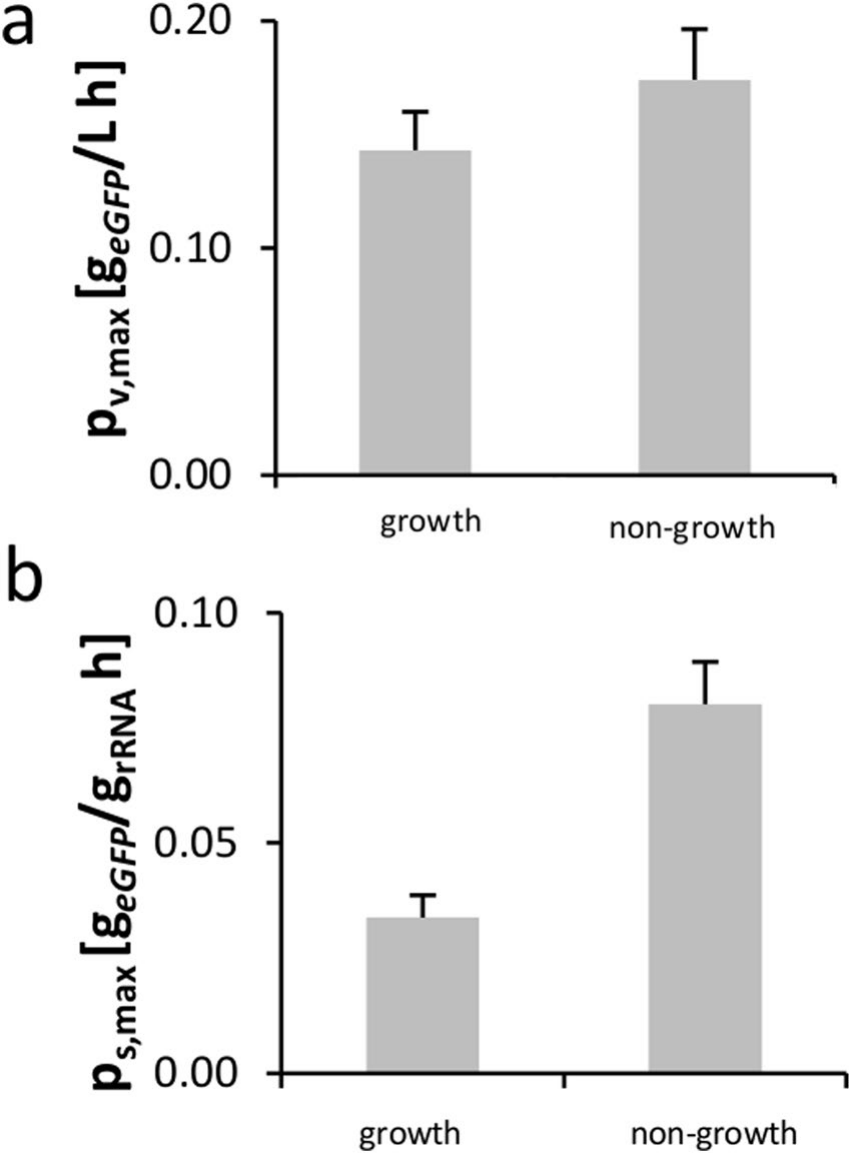
Performance characteristics of cell-free expression systems derived from growing and non-growing cells. (**a**) Maximum volumetric synthesis rates at cell-free expression of eGFP. (**b**) Maximum specific synthesis rates.

**Figure 5:**
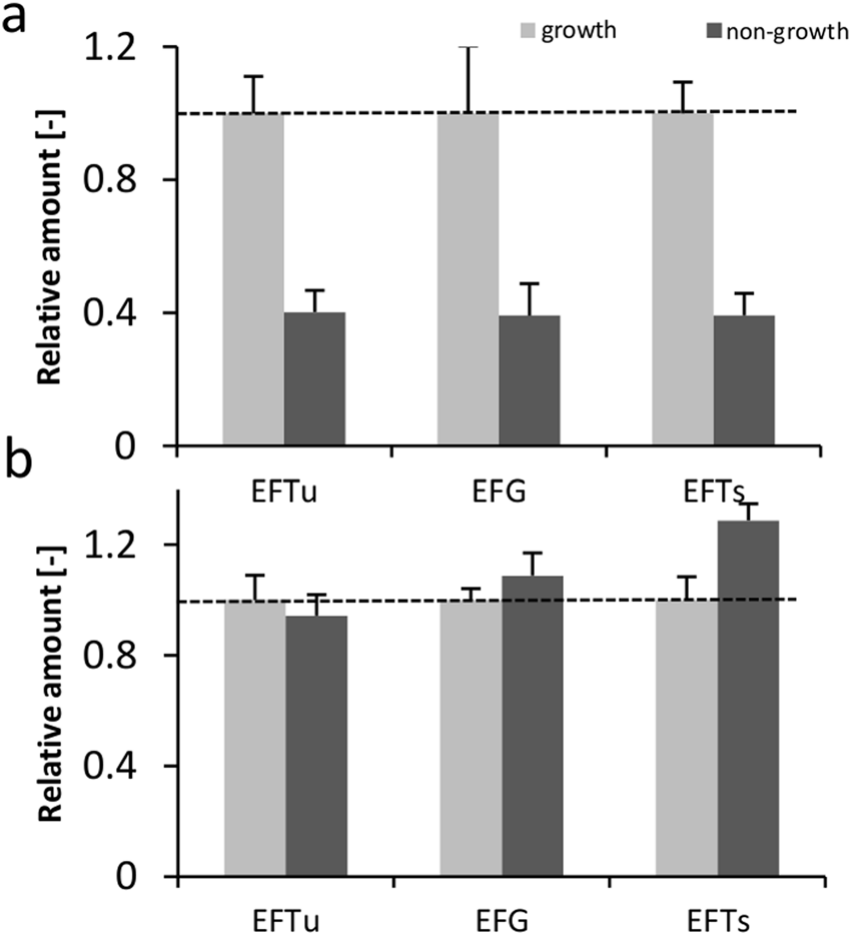
Characterization of the translation machinery. (**a**) Relative amount of elongation factors in cell-free extracts derived from stressed cells (non-growth) compared to those derived from non-stressed conditions (fast growth). (**b**) Relative amount of elongation factors normalized by the ribosome content (ribosomal protein S1). Shown are means and standard deviations of two peptides per elongation factor from two independently prepared cell-free extracts.

We further analyzed the performance of our cell-free translation systems by calculating volumetric and specific protein production rates, P_v,max_ (g_eGFP_/L h) and P_S,max_ (g_eGFP_/g_rRNA_ h), respectively. We found P_v,max_ to be approximately 0.15–0.2 g_eGFP_/(L h) for all conditions tested (Fig. 4a). We observed higher specific production rates in the *in vitro* translation system from non-growing cells (Fig. 4b), leading us to consider whether this phenomenon could be explained by alterations of the translation machinery. As previously shown in our lab, stoichiometric changes in the translation apparatus can result in increased overall translation rates^42^. In detail, increased ternary complex concentrations (e.g., the elongation factor Tu) can increase CFPS synthesis performance. Therefore, we used a relative proteomics approach to detect possible changes in the stoichiometry between ribosomes and elongation factors in the lysates. The proteomic workflow involved a ^13^C-labeled reference lysate^19^ to account for variations in the sample preparation, such as tryptic digestion. Two peptides per elongation factor were measured, and their relative amount was compared in lysates from growing and non-growing cells (Fig. 5a).

In lysates from non-growing cells, the amount of elongation factors was reduced. While the fate of ribosomes at growth arrest has been extensively studied, not much is known how other components of the translation machinery are affected. Our result suggests that elongation factors follow a similar phenomenon as that detected for ribosomes. Moreover, this result connotes that the given stoichiometry of ribosomes and elongation factors could be affected under non-growth conditions. To clarify this issue, relative amounts of elongation factors were normalized by the ribosomal protein S1 (RPS1) content, which is a measure of the ribosome concentration in the extracts (Fig. 5b). Comparison of normalized elongation factors revealed an unaltered ratio of ribosomes to EFTu and EFG, respectively, whereas the ratio of EFTs to ribosomes was slightly higher (*p* < 0.05). It can be hypothesized that the translation machinery in lysates from resting cells benefits from the higher concentration of EFTs; however, it is unlikely that this results in the high specific translation rates calculated above.

At first glance, our observation contradicts the current agreement that a high growth rate results in high active cell-free extract, and a low growth rate results in low active cell-free extract, as demonstrated earlier^30^. However, our experimental strategy differs from this previous work, where fed-batch fermentations were used to enable growth-limiting conditions (slow growth) by controlling the glucose availability. A proceeding carbon limitation results in numerous cellular long-term adaption and regulation phenomenons, such as the stringent response, which results in a reduced synthesis of ribosomal proteins^43^. In addition, second messengers (alarmones), such as, ppGpp, cAMP or FbP accumulate or respectively deplete in the cell at these carbon-limited proceedings, termed as ‚hunger‘ scenario (for a review see Ferenci *et al*.^44^). Thus, a gradual fluctuation of metabolic stressors or global regulators such as Cra, Crp or RelA/SpoT, may occur^43,45,46^. While we did not measure the levels of alarmons such as ppGpp in our extracts, the obviously low levels of sigma factor 38 (see below), that is induced by ppGpp^47^ let us assume that the stringent response was not triggered in our cultivation scheme. Moreover, we were able to detect residual glucose after ‘overnight’ cultivation that was most likely not fully consumed due to the observed drift in pH. Hence, as reported earlier^45^, it is obvious that the physiological response of *E. coli* to glucose limitation (mineral medium) during fed-batch is different to that at the very end of a batch cultivation, for example characterized by a carbon-starved stationary phase (which is termed ‚starvation’, apart from the above mentioned ‚hunger’ phase according to Ferenci *et al*.^44^). In contrary to a fed-batch scenario, our cells showed unlimited growth and almost maximum growth rates (µ ~ 1 h^−1^) during the exponential growth phase on complex media. After this initial period of maximum growth, the cells were harvested at non-growth (stationary phase). We conclude that even though a growth arrest drastically impacts the cell, the stoichiometry between ribosomes and elongation factors is conserved and ribosomes remain active.

Next, we aimed to compare the amount of ribosomes actively participating in the translation reaction in both cell-free extracts. As ribosomes coupled with mRNA can be regarded as actively translating, our experimental strategy was to estimate the amount of eGFP mRNA associated with ribosomes. Hence, cell-free reactions were quenched in liquid nitrogen. The reactions were analyzed on sucrose gradients and the fraction containing polysomes, monosomes, and ribosomal subunits were collected (Supplemental Fig. S5). Total RNA was extracted and the eGFP mRNA was relatively quantified by qPCR. For a better comparison, the eGFP mRNA amount obtained from the ribosome fraction was normalized by the total rRNA amount. This normalization was possible because we uniformly found approximately 15% of total ribosomes in polysomes. We calculated a higher ratio of mRNA per rRNA in lysates originating from non-growing cells (Supplemental Fig. S5). Therefore, it appears that a larger percentage of total ribosomes are active in the lysate derived from cells harvested at non-growth. More broadly, this result suggests that only a certain amount of ribosomes can be recruited to actively participate in our CFPS system. This hypothesis was emphasized by the fact that a reduction of the amount of lysate (from growing cells) in the reaction resulted in similar overall production rates (0.14 g_eGFP_/L h). Earlier reports have shown a fraction of actively translating ribosomes varying between 22% and 72%^30,48^. Recently, a measurement of ribosome activity in CFPS using an arrest peptide demonstrated an active ribosome fraction of only 40%^49^. Therefore, it is evident that in current CFPS systems only a fraction of the total ribosome content is actively participating in translation. It can be speculated, that in the CFPS system derived from non-growing cells, certain mechanisms associated with this specific physiological background favor a higher fraction of ribosomes contributing to peptide synthesis. To unravel whether this highly effective translation is performed by ‘specialized ribosomes’ (for a review on that topic refer to Xue *et al*.^50^) is a challenge for future investigations.

Although it can be suggested that the potential of our CFPS system has not yet been fully exploited, our reported volumetric production rates (0.2 g/L h for large scale and 0.35 g/L h for small scale reactions) already compete with current CFPS systems using similar scales^51,52^. Nevertheless, our group is currently establishing an automation platform for high-throughput of CFPS to address optimization of the system.

The slow release of glucose from glycogen or starch can initiate long-lasting *in vitro* reaction systems by activating the glycolytic pathway^21^. Accordingly, we tested the potential of using endogenous energy regeneration systems in the lysates originating from non-growing cells. Therefore, *in vitro* translation reactions were performed in the presence of glycogen. We found active protein synthesis over several hours, demonstrating that the glycolytic pathway is fully active (Supplemental Fig. S6). This is in accordance with previous reports, which state that an increased synthesis of glycolytic enzymes was observed in the stationary phase^53^.

In essence, the translation machinery in cell-free extracts from non-growing, stationary cells is intact and enables synthesis of proteins *in vitro*. This holds also true for another strain, (*E. coli* MG1655) that was tested (Supplemental Fig. S7). Moreover, this approach doubles the amount of obtained cell-free extract per cultivation. Furthermore, cultivation of biomass for extract preparation can be simply performed over night.

### Conditioning of the biomass activates parallel transcription

Given that ongoing growth is not a constraint for active cell-free extract, we sought to exploit the potential of this expression system to test transcription by alternative σ factors. Thus, we aimed to expand the repertoire of possible transcription activation units. Transcription by the RNA core polymerase is dependent on binding of a σ factor to enable promoter recognition and transcription initiation. Although the majority of genes are transcribed by RNAP and housekeeping σ^70^, 6 “alternative” σ factors are available, which enable the expression of a specific set of genes^54^. These alternative σ factors are expressed at changing environmental conditions, which are usually related to cellular stress, such as starvation or heat. Hence, this experimental approach also allows characterizing regulatory mechanisms associated with non-growth.

**Figure 6:**
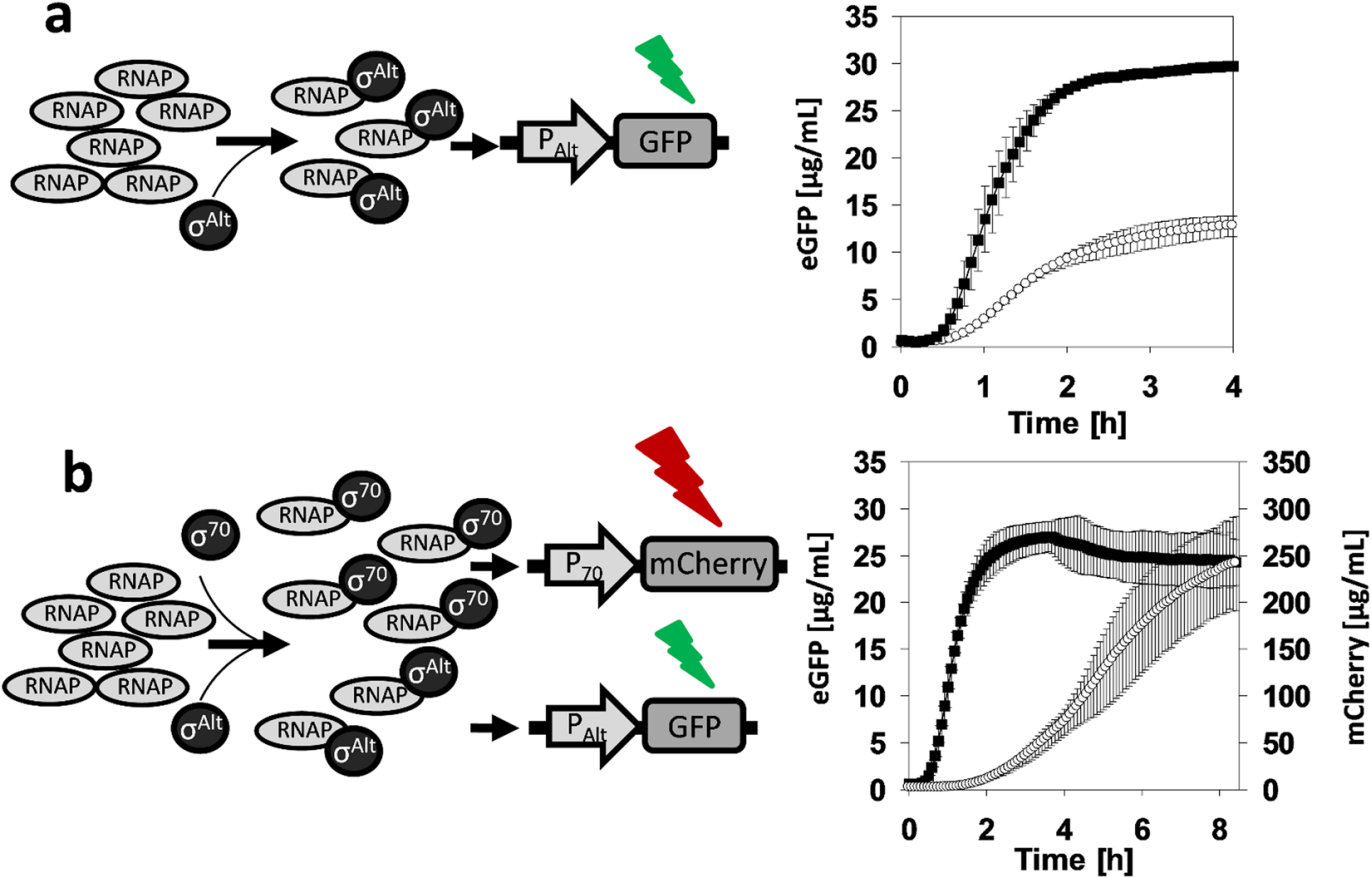
Activation of parallel transcription. (**a**) Transcription and translation systems based on an “alternative” σ^32^ and *E. coli* core RNA polymerase. Transcription of eGFP mRNA was under control of a promoter specific to σ^32^. Cell-free protein synthesis of eGFP derived from stressed biomass (▪) shows elevated activation of σ^32^ compared to the control (○), n = 3. (**b**) Parallel transcription with a high and low translation output signal, by simultaneously activation of σ^32^ (▪), which controls expression of eGFP, and σ^70^ (○), which controls expression of mCherry, n = 3.

σ^38^ controls many genes that are induced at onset of the stationary phase or as a response to high osmolarity^55^. Thus, we tested if it is possible to recruit σ^38^ for direct transcription in our *in vitro* translation system derived from non-growing cells. We chose to use the promoter of *osmY* to activate transcription of eGFP *in vitro*. This promoter has previously been used for designing genetic circuits^2^. We observed only a basal expression of eGFP through the σ^38^ activation unit under all conditions tested (Supplemental Fig. S8). We reasoned that not enough σ^38^ was present in the cell-free extract at the point of harvest. Even though we detected a down regulation of ribosomes in cell extract derived under non- growth (resting) conditions, this result suggests that certain regulation mechanisms associated with the stationary phase were not fully active yet. Hence, we screened for other σ factors and assessed the potential of recruiting heat shock σ^32^ to activate transcription. The biomass of *E. coli* A19 was exposed to heat stress (45 °C) for 30 min, after which it was rapidly chilled on ice and the cell-free extract was prepared. No growth was observed during heat exposure indicating that cells were stressed and stationary. For expression by σ^32^, we cloned the strong heat shock promoter of *dnaK* in front of GFP^56^. To validate the expression system, we performed *in vitro* translation reactions with cell-free extracts from stressed (heat) and non-stressed (control) cells. Compared to that of the control (6 µg/mL h), an approximately four-fold higher synthesis rate was obtained for the translation systems derived from stressed cells (23 µg/mL h) (Fig. 6a). This clearly demonstrates the proof-of-principle of recruiting alternative σ factors to enlarge the transcription machinery *in vitro*.

Subsequently, a parallel transcription unit was designed by engaging more than one σ factor. Our goal was to create a resistor-like network consisting of a high-resistance unit that gives a low-signal output and a low-resistance unit that gives a high-signal output. This was obtained by simultaneously recruiting σ^70^ for the expression of mCherry and σ^32^ for the expression of eGFP (Fig. 6b). At saturating plasmid concentrations (12 nM) we obtained two distinct output signals (final titers) that differed approximately by one order of magnitude. From Fig. 6b it can be seen that a different kinetic was found for the expression of mCherry compared to that of eGFP. However, this is more likely owing to a long maturation time of the mCherry chromophore^57^ than to differences in the expression pattern. This phenomenon was validated by the eGFP expression from σ^70^, that gave a similar titer as obtained by the expression of mCherry, but showed a fast expression kinetic (Supplemental Fig. S9). This *in vitro* expression platform allows the precise channeling of the translation capacity to generate more than one output of a specific signal height without adjusting the DNA template concentration. In principle, this allows the design of artificial multi-enzyme reaction cascades (metabolic networks) where certain stoichiometric quantities of enzymes are required to meet an optimal reaction flux.

## Conclusion

To generate high-active cell-free extracts, it is generally considered preferable to maintain high-active translation modules. Hence, cell harvest is conducted at logarithmic growth. Here, we demonstrated the activity of a cell-free expression system derived from stressed cells by late harvest of the biomass at the stationary growth phase. Moreover, we were able to demonstrate that the stoichiometry of ribosomes and translation factors was conserved. Consequently, we conclude that the ribosomes and associated proteins, such as elongation factors, that are remaining in the cells at the stationary phase are highly active and allow *in vitro* translation. However, while it seems that the reduced amount of ribosomes in cell-free extracts derived from stationary cells are efficiently used for cell-free translation; we found evidence that only a partial fraction of ribosomes in cell-free extract from exponentially growing cells participate in translation. Looking forward, the identification of the principal mechanism behind this limitation is a challenging task that will be pursued.

This finding gave us the opportunity to create lysates with other physiological backgrounds, namely, from stress, non-growing conditions. As the cell reacts to certain stress conditions by expressing alternative σ factors, we investigated whether such a transcription factor can be recruited in hopes to expand the repertoire of genetic transcription units and to gain insights into the complex regulatory mechanisms that are linked to growth arrest. Although it was demonstrated that the stationary phase σ factor (σ^38^) in combination with a promoter derived from *osmY* did not enable efficient protein expression, most likely because σ^38^ was not efficiently upregulated or its affinity to the used promoter was poor, conditioning of the biomass via heat-shock enabled protein expression via σ^32^. This clearly demonstrated that non-growing, stressed *E. coli* cells provide a functional environment for *in vitro* translation and are a highly versatile tool for cell-free biology.

In summary, our advances show the growing potential of cell-free translation systems to design more complex *in vitro* systems.

## Methods

### Biomass cultivation and cell-free extract preparation

*E. coli* A19 and MG1655 were cultivated in 2-L shaking flasks in 2 × YTPG medium (10 g/L yeast extract, 16 g/L tryptone, 5 g/L NaCl, 18 g/L glucose), 2 × YTP medium (no glucose), or mineral medium as described previously^36^. Cells were harvested during the exponential or stationary phases by placing the shaking flasks in an ice bath. The biomass was collected by centrifugation (8000 × *g* for 20 min at 4 °C). The following steps were performed as described previously^23,24^. The biomass was resuspended in S30 buffer (14 mM magnesium acetate, 60 mM potassium acetate, 10 mM Tris, pH 8.0, 2 mM DTT) by adding 1 mL of buffer per gram of biomass. The biomass was lysed using high-pressure homogenization (EmulsiFlex-C5, Avestin, Canada) at 12,000 kPa. The cell debris was removed by two centrifugation steps at 30,000 × *g*, each for 30 min, at 4 °C. A run-off reaction was performed by incubating the cell-free extract for 80 min at 37 °C. The extract was dialyzed against a 100 times larger volume of dialysis buffer (S30 buffer) for 4 h at 4 °C. The cell-free extract was then centrifuged at 4000 × *g* for 20 min at 4 °C, and finally aliquoted and stored at −70 °C. For heat shock experiments, the biomass was placed in a water bath (45 °C) for 30 min before being stored.

### Cell-free reaction mixture preparation

The cell-free reaction mixture consisted of 80 mM HEPES-KOH (pH 8.0); 1.2 mM ATP; 1 mM GTP, CTP, and UTP; 2 mM DTT; 90 mM potassium glutamate; 20 mM ammonium glutamate; 14–18 mM magnesium glutamate; 34 µg/mL folinic acid; 1 mM 20 amino acids; 2% PEG (8000); 60 mM creatine phosphate; 240 µg/mL creatine kinase; 3 U/µL T7 RNA polymerase (Roche Diagnostics, Mannheim, Germany); and 24% (v/v) cell-free extract. The total reaction-mixture volume was 250 µL. The magnesium concentration was optimized for each batch of cell-free extract. For cell-free reactions with glycogen (2% w/v), NAD (0.3 mM) and CoA (0.26 mM) were added.

### Ribosome and polysome profile analysis

Cell-free extract samples were placed in 5–20% (w/v) sucrose gradients with 10 mM Tris-HCL, pH 8.0, 12 mM magnesium acetate, 100 mM NH_4_CL, and 1 mM DTT. Cell-free reaction samples were quenched in liquid nitrogen and placed in 10–40% (w/v) sucrose gradients. All samples were centrifuged in an SW41 Ti rotor (Beckman Coulter, Brea, USA) at 30,000 rpm (170,000 × *g*) for 3 h at 4 °C. The gradients were analyzed by pumping them through a UV cuvette and measuring their absorbance at 260 nm.

### qPCR analysis

For relative quantification of mRNA associated with ribosomes, sucrose gradients were collected and RNA was extracted by LiCl. Briefly, 0.4 volumes of 9 M LiCl were added to RNA-containing fractions of sucrose gradients. Moreover, 100 µL of MS2 RNA, which serves as an internal reference, was added to all samples. RNA was precipitated at −20 °C over night. The samples were centrifuged (20,000 × *g*, 30 min, 4 °C) and the RNA-containing pellet was washed twice with 70% (v/v) ethanol. qPCR was performed using the *Power* SYBR® Green RNA-to-CT™ 1-Step Kit (Thermo Scientific) in a RealPlex cycler (Eppendorf). Primers for qPCR analysis were designed using Primer Express 3.0 software (Applied Biosystems). Primer sequences (5′ to 3′) for qPCR of EGFP mRNA: forward CTGCTGCCCGACAACCA, reverse TGTGATCGCGCTTCTCGTT; primer sequences (5′ to 3′) for qPCR of MS2 RNA: forward GCTCTGAGAGCGGCTCTATTG, reverse CGTTATAGCGGACCGCGT. The PCR efficiency was 1.01. We calculated the ΔCT of eGFP mRNA and MS2 RNA between samples to determine the mRNA amount associated with translating ribosomes.

### rRNA quantification and analysis

Extraction and quantification of rRNA was performed as described previously^19,58^. Briefly, RNA-containing samples (20 µL) were mixed with MS2 RNA (20 µL) and 0.5 mL extraction buffer (10 mM Tris-HCl [pH 8.0], 10 mM NaCl, 1 mM sodium citrate, and 1.5% [w/v] SDS). The samples were mixed with 250 µL ice-cold NaCl (saturated solution), incubated on ice for 10 min, and the derived protein-SDS-DNA precipitate was collected by centrifugation (20,000 × *g*, 20 min, 4 °C). The clear supernatant (500 µL) was transferred to fresh tubes and ethanol (1 mL) was added to precipitate RNA. After incubation of the samples at −70 °C for 1 h, the precipitated RNA was collected by centrifugation (20,000 × *g*, 30 min, 4 °C) and washed with 1 mL ice-cold ethanol (70%). The samples were dried and resuspended in RNase-free water prior to analysis. Distinct rRNA species were measured using capillary gel electrophoresis, with laser-induced fluorescence detection, using an Agilent 2100 Bioanalyzer. 16 S and 23 S rRNA were quantified based on the added internal standard.

### Mass spectrometry

Relative quantification of elongation factors by liquid chromatography-quadrupole time-of-flight-MS was executed as described previously^19,59^. Samples were mixed with an equal volume of a stable isotope-labeled (^13^C_6_) cell-extract that served as a reference. Next, in-solution trypsin digestion of proteins was performed overnight. For MS analysis, samples were brought to a final concentration of 5% acetonitrile and 1% formic acid.

### Plasmids

The plasmid pJOE4056.2 was used for the cell-free expression of eGFP from the T7 promoter. Plasmid pBEST-OR2-OR1-Pr-UTR1-deGFP-T500^15^, which was a kind gift from Vincent Noireaux (Addgene plasmid # 40019), was used for cell-free expression from the core RNA polymerase. For expression by RNAP and σ^70^, eGFP from pBEST-OR2-OR1-Pr-UTR1-deGFP-T500 was replaced by mCherry after digestion with *NheI* and *XhoI*. For expression by RNAP and σ^38^ and σ^32^, the Pr promoter from pBEST-OR2-OR1-Pr-UTR1-deGFP-T500 was replaced by the promoter from *osmY* (specific to σ^38^) and *dnaK* (specific to σ^32^), respectively, using restriction enzymes *SphI* and *NheI*. All constructs were checked by sequencing to assure accuracy.

### Fluorescence measurements

*In vitro* synthesis of eGFP in a scale of 250 µL was monitored online by fluorescence detection (excitation filter 485 nm; emission filter 520 nm) in a Synergy 2 plate reader (Biotek Instruments, USA) at 37 °C. For downscaling the reactions, the synthesis was performed in PCR tubes. mCherry was measured at 540 and 590 nm. Quantification of eGFP and mCherry was performed by comparison to a standard.

### Data availability

All data generated or analyzed during this study are included in this published article (and its Supplementary Information files).

## Electronic supplementary material

Supplementary Information
