# Supplementary material for: Cell-free protein synthesis from non-growing, stressed Escherichia coli
Source: Sci Rep. 2017 Nov 28;7:16524. doi: 10.1038/s41598-017-16767-7 (PMC5705671; doi:10.1038/s41598-017-16767-7)
Supplement: Supplementary file 1 — Supplementary Information [file 41598_2017_16767_MOESM1_ESM.pdf]

# Supporting Information

## Cell-free protein synthesis from non-growing, stressed *Escherichia coli*

Jurek Failmezger, Michael Rauter, Robert Nitschel, Michael Kraml and Martin Siemann-Herzberg*

Institute of Biochemical Engineering
University of Stuttgart
Stuttgart, Germany

*Corresponding author
siemann@ibvt.uni-stuttgart.de (MSH)

**Content:**

Figure S1: Scale down.

Figure S2: rRNA integrity.

Figure S3: Ribosome profiles.

Figure S4: Expression based on cell-free extract from cells cultivated in 2 x YTP media.

Figure S5: Quantification of ribosomes associated with mRNA.

Figure S6: Cell-free protein synthesis from non-growing, stationary cells fueled with glycogen.

Figure S7: In vitro translation with extracts derived from non-growing *E. coli* MG1655

Figure S8: Cell-free translation reactions with $\sigma^{38}$.

Figure S9: Cell-free translation reactions with $\sigma^{70}$.

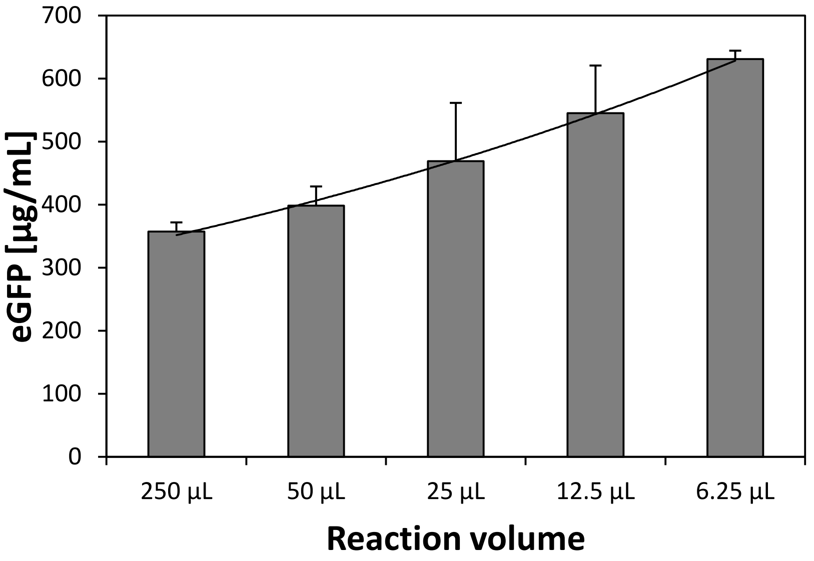


Figure S1: Scale down of the cell-free translation reaction.

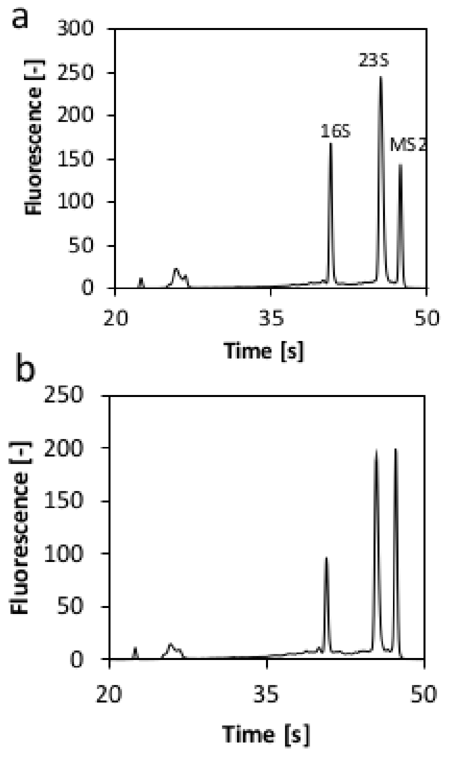


Figure S2: Analysis of rRNA integrity. rRNA from cell-free extract originating from fast growing cells (a) and from cells harvested at the stationary phase (b) was extracted and analyzed by CGE-LIF. MS2 RNA denotes the internal standard that is added for quantification.

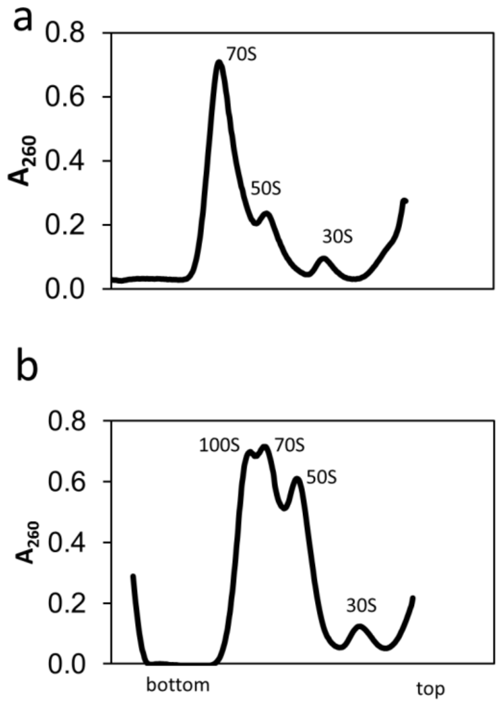


Figure S3: Ribosome profile analysis of cell-free extracts derived from stationary cells cultivated on 2 x YTP medium (a) and mineral medium (b).

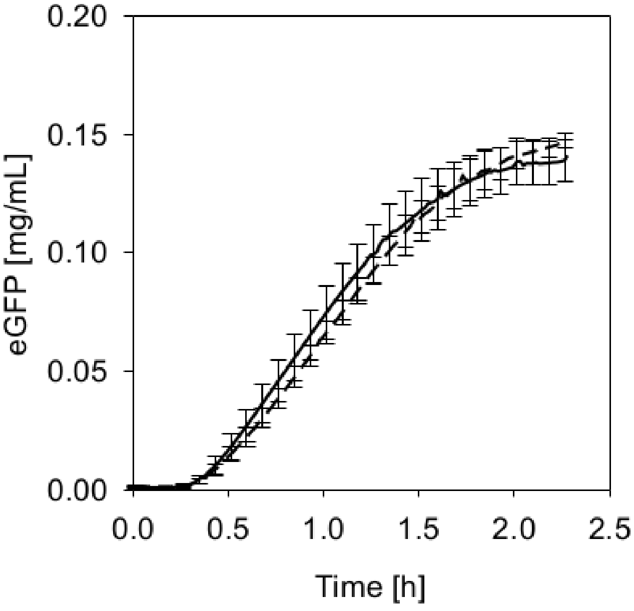


Figure S4: Expression based on cell-free extract of cells cultivated in 2 x YTP media. The solid line denotes extract derived from stationary cell and the dotted line from fast-growing cells.

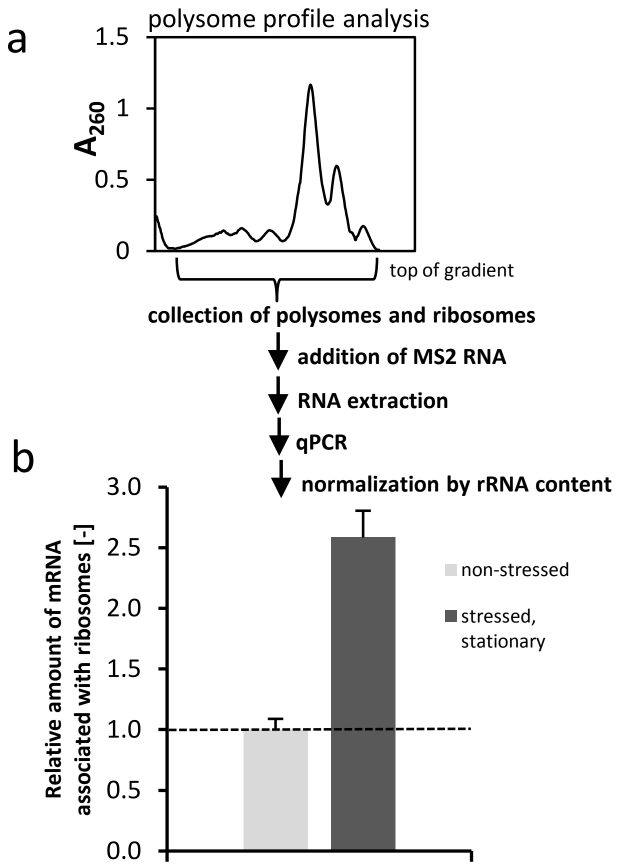


Figure S5: Analysis of ribosomes associated with mRNA. (a) mRNA in the polysome fraction was extracted and quantified by qPCR. (b) Comparison of the relative mRNA amount that was associated with ribosomes in cell-free translation reactions derived from growing, non-stressed and non-growing, stressed cells.

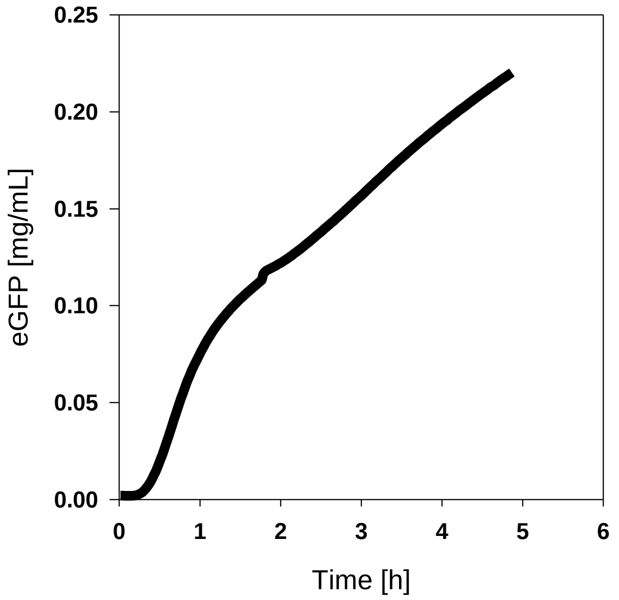


Figure S6: In vitro translation reaction performed with a cell-free extract from non-growing cells fueled with glycogen.

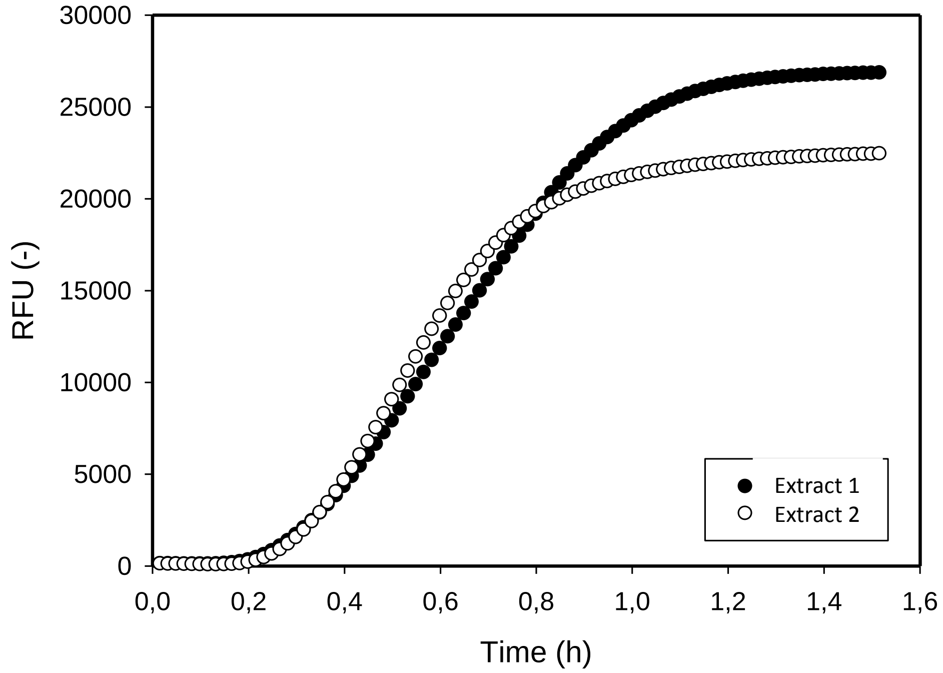


Figure S7: In vitro translation with extracts derived from non-growing *E. coli* MG1655. Shown are two independently prepared extracts derived from non-growing *E. coli* MG1655, that demonstrate the potential of eGFP synthesis.

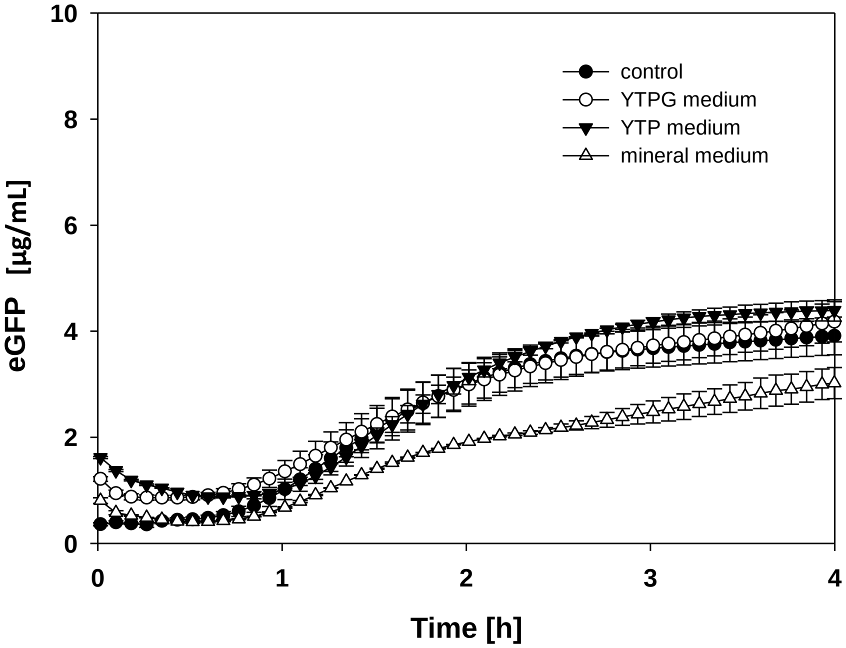


Figure S8: Cell-free translation reactions with $\sigma^{38}$ and cell-free extracts from various cultivation conditions, namely, fast growth (control) and non-growth (cultivation on YTPG, YTP, and mineral media) conditions.

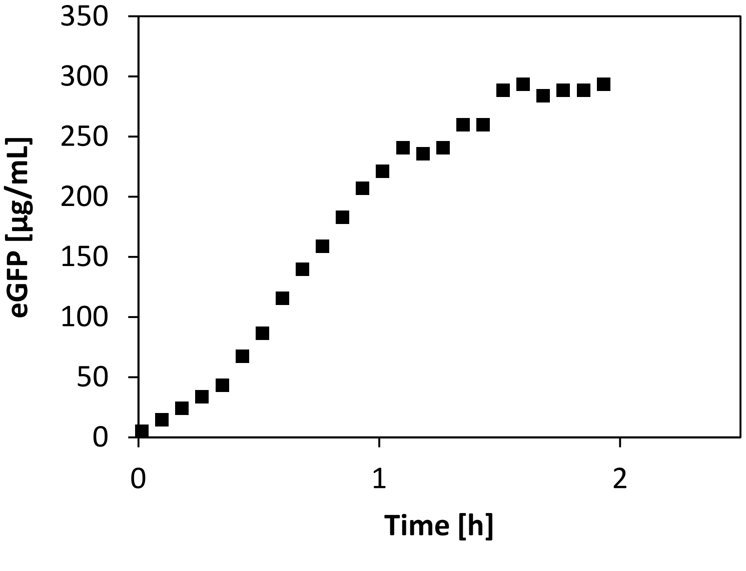


Figure S9: Cell-free expression of eGFP from $\sigma^{70}$ and cell-free extract from heat shocked biomass.
